# Clinico-epidemiological Characteristics of Corrosive Ingestion: A Cross-sectional Study at a Tertiary Care Hospital of Multan, South-Punjab Pakistan

**DOI:** 10.7759/cureus.2704

**Published:** 2018-05-29

**Authors:** Muhammad Usman Hashmi, Mansoor Ali, Kaleem Ullah, Abdul Aleem, Iftikhar H Khan

**Affiliations:** 1 Thoracic Surgery, Nishtar Medical University Hospital, Multan, PAK; 2 Upper Gi and Thoracic Surgery, Nishtar Medical University Hospital, Multan, PAK; 3 Thoracic and General Surgery, Nishtar Medical University, Multan, PAK

**Keywords:** corrosive intake, caustic ingestion, clinico-epidemiological features, chemical burn, household product poisoning

## Abstract

Introduction

Corrosive ingestion is a grave public health problem. It is a medical emergency and shows diverse clinical presentations. The ingestion of corrosive substances has devastating effects on upper gastrointestinal and respiratory tracts and the corrosive injury is associated with numerous life-threatening complications. The present study aims to explore the clinico-epidemiological characteristics of patients of corrosive ingestion presenting at a tertiary care hospital of Multan, Pakistan.

Method

The target study population consists of all the patients with primary diagnosis of corrosive ingestion who presented to the department of thoracic surgery, Nishtar Medical University Hospital Multan, Pakistan, from January 2016 to December 2017. The follow-up cases and the cases with ingestion of substances other than corrosives were not included in the study. All the included cases were evaluated by detailed history, thorough physical examination and the necessary investigations. The post-cor­rosive tissue damage was classified accord­ing to Zargar’s classification system. All the demographic data and other variables were measured and recorded using a Performa. The data were analyzed by using computer program SPSS 21 version.

Results

The total study population was 206 patients. There were 135 females (65.5%) and 71 male patients (34.5%). Age ranged from 2 to 42 years (mean 23.44 ± 7.19). Only seven cases were found in the age group of 2-7 years. The residents of rural areas showed a slightly increased inclination towards corrosive ingestion. One hundred and ten cases were unmarried (53.4%) while 90 patients were married (43.7%). The incidence of corrosive ingestion was much high in illiterate/less educated patients belonging to the groups of lower socio-economic status. One hundred and ninety-seven patients ingested corrosive substances deliberately with the suicidal intention (95.6%). The acid used as bathroom cleaner and the laundry bleaches were the most commonly used corrosive agents. In 166 cases the corrosive materials were already present at home for domestic purposes (80.6%), but 18 subjects particularly purchased these corrosive substances to commit suicide. The quantity of ingested material ranged between 10 ml and 150 ml with a mean of 42.6 ml ± 33.2. The shortest hospital stay was one day, and the longest one was 60 days. Esophagus and oropharyngeal area were the most common site which sustained the corrosive injury, whereas corrosive injury to duodenum was least frequent (34.5 %).

Conclusion

Corrosive ingestion is a serious medical problem and it requires a multidisciplinary approach and a good coordination between different medical specialists. Underprivileged teenager females of rural areas are more likely to ingest corrosive materials with suicidal intention. In most of the ingestions, household cleaning products are used. Only the patients with severe corrosive injury should be admitted to intensive care units. Enforcing regulations for the manufacturers of household cleaning products can significantly reduce the incidence of this potentially fatal condition.

## Introduction

Corrosive ingestion is a grave public health problem across the globe [1]. It is more common in the developing countries, but still seen in developed countries. Even in the United States about 5,000 to 15,000 corrosive ingestions are reported per year [2-4]. Annually, more than 40,000 cases of corrosive ingestion are reported in England and Wales [5]. Corrosive ingestion is a medical emergency and shows diverse clinical presentations. Its clinical course is exceedingly complex. Corrosive ingestions may result in extensive injury to the lips, oral cavity, pharynx, and the upper airway. It may cause massive hemorrhage, gastrointestinal tract perforation, aorto-enteric/gastro-colic fistulae, tracheal stenosis, and the tracheoesophageal fistula [6]. Corrosive agents badly damage the esophagus [4]. They can cause esophageal perforation, stricture formation and later may lead to the development of esophageal carcinoma [7]. The cumulative rate of these various complications is estimated up to 23.61-89.3% [8, 9]. The extent of corrosive injuries is mainly dependent upon the exposure time, nature, amount and concentration of the corrosive agent [10]. Generally, acids with pH less than 3 or bases with pH greater than 11 are notorious for causing corrosive injury of tissues [1]. The examples of most commonly used corrosive materials include sulphuric acid, nitric acid, phosphoric acid, hydrochloric acid, oxalic acid, sodium hydroxide, potassium hydroxide and bleaches [7]. These corrosive materials are found in drain cleaners, various cleaning agents, hair relaxers, dishwasher detergents, and disk batteries [7].

Recent medical literature reveals that corrosive ingestion is seen in every age group. Ingestion may be either deliberately with suicidal intent or accidental. In developing countries, the incidence of corrosive ingestion is significantly higher and in most cases remains unreported. The real prevalence cannot be deduced from randomly scattered observations and personal judgments of healthcare professionals [11]. The lack of safe “child-proof” containers, unregulated access to corrosive agents, cultural specific inclination to ingest corrosives for suicidal purpose, the absent or scarce standard medical care in rural areas, malnourishment and loss of follow-up among survivors are the main challenges which needs to be addressed in a focused manner [3, 12]. Medical literature regarding epidemiology and clinical presentation of corrosive ingestion patients is inadequate. A study by World Health Organisation (WHO) stated that in past 17 years, only 37 papers were published concerning corrosive ingestion in low- and lower-middle income countries. Out of these 37, only eight papers focused the specific epidemiology of corrosive ingestion [13].

Nishtar Medical University Hospital is a tertiary care health facility in Multan, South Punjab, Pakistan. Its Department of Upper GI and Thoracic Surgery is serving the patients of this region for the last two decades. Our department also receives referrals from the various other hospitals of adjacent areas and even from other provinces. Hence, its catchment population is estimated at 4.5 million. Currently, we observed a very high incidence of corrosive ingestion in various areas of South Punjab, and it was causing a huge burden on our department. We conducted this study as it was vitally important to study and explore the causative factors of this public health problem. Our work aims to analyze the clinico-epidemiological characteristics and the severity of injury among the patients of corrosive ingestion.

## Materials and methods

This prospective descriptive cross-sectional study was conducted at the Department of Upper GI and Thoracic Surgery, Nishtar Medical University Hospital Multan, Pakistan. The duration of the study was from June 2016 to May 2017. All the cases with the primary diagnosis of corrosive ingestion were included in the study. The inclusion criterion was patient with acute corrosive ingestion, hospitalized in the department of upper GI and thoracic surgery. Only new cases were included, follow-up cases and cases with ingestion of substances other than corrosives were not included in the study. All these cases were evaluated by detailed history and thorough physical examination. As per standard operative principles, patients in the acute phase were hospitalized in intensive care unit and urgent upper GI endoscopy was performed. The post-corrosive tissue damage was classified according to ZARGAR’s classification system for caustic injury. Table 1 contains the information for this classification system.

**Table 1:** Zargar's grading classification of the mucosal injury caused by ingestion of caustic substances.

Grade	Features
Grade 0	Normal
Grade 1	Superficial mucosal edema and erythema
Grade 2	Mucosal and submucosal ulcerations
Grade 2A	Superficial ulcerations, erosions, exudates
Grade 2B	Deep discrete or circumferential ulcerations
Grade 3	Transmural ulcerations with necrosis
Grade 3A	Focal necrosis
Grade 3B	Extensive necrosis
Grade 4	Perforations

Injury to the epiglottis and larynx was assessed by laryngoscopy. A team of otorhinolaryngologists, pulmonologists and gastroenterologists also examined these patients. According to needs of patients, various laboratory investigations and imaging studies (e.g., radiographs, contrast studies, computed tomography (CT) scanning, and upper GI endoscopy) were performed for the further evaluation. We interviewed all the cases for the measurement of different variables such as age, gender, type and amount of ingested agent, the cause of ingestion (intentional or accidental), prominent symptoms, and past history of any psychiatric ailments. The approximate quantity of ingested corrosive agent was determined from the data which was carefully gathered during history taking from the patients and/or their families. These pieces of information were recorded on a specifically designed Performa. All statistical analysis was done in IBM SPSS Statistics for Windows, Version 19.0 (Released 2010, IBM Corp., Armonk, NY). The mean and the standard deviation were computed for numerical variables like age. Frequencies and percentages were computed for categorical variables, such as gender, type of ingested agent, the cause of ingestion, prominent symptoms, and the sites of post-corrosive ingestion injuries.

## Results

The current study comprised of 206 cases. There were 135 females (65.5%) and 71 male patients (34.5%). Age ranged from 2 to 42 years (mean 23.4 ± 7.2). Only seven cases were found in the age group of 2-7 years. Residents of rural areas showed a slightly higher inclination towards corrosive ingestion. One hundred and nine cases belonged to rural areas (52.9 %) and 97 patient were from urban areas (47.1 %). One hundred and ten cases were unmarried (53.4%) while 90 patients were married (43.7%). The incidence of corrosive ingestion was much high in underprivileged population, belonging to lower socio-economic status. Similarly, corrosive ingestion was much frequent among illiterate and less educated patients. Past history of psychiatric diseases was reported in 43 cases (20.9%). One hundred and ninety-seven patients ingested corrosive substances deliberately with the suicidal intention (95.6%). Nine cases ingested corrosive material accidentally (4.4 %). Of these nine cases, just two were adult, rests of them were children. Table 2 shows the demographic characteristic of all the patients.

**Table 2:** Demographic characteristics of all patients.

Variable	Frequency	Percentage
Age (Years)		
02-7	7	3.4%
15-30	165	80.1%
30-42	34	16.6%
Gender		
Female	135	65.5%
Male	71	34.5%
Education		
Illiterate	49	23.8%
Primary or less	62	30.1%
Middle	33	16.0%
Matriculate	43	20.9%
Intermediate	15	7.3%
Graduation	4	1.9%
Residential area		
Urban	97	47.1%
Rural	109	52.9%
Marital status		
Single	110	53.4%
Married	90	43.7%
Separated	4	1.9%
Divorced	2	1.0%
Monthly family income (Pakistani rupees)		
1000–5000	35	17.0%
5000–10,000	73	35.4%
10,000–15,000	56	27.2%
15,000–20,000	26	12.6%
20,000–40,000	16	7.8%
Religion		
Muslim	203	98.5%
Christian	3	1.5%
Past history of psychiatric ailment		
No history	163	79.1%
Positive history	43	20.9%
Intention		
Suicidal	197	95.6%
Accidental	9	4.4%

The acid used as bathroom cleaner and the laundry bleaches were the most commonly used corrosive agents. The relative frequency of use of bathroom cleaner acid and laundry bleaches was 129 (62.6%) and 44 (21.4%), respectively. In 166 cases the corrosive materials were already present at home for the domestic purpose (80.6%), but 18 subjects particularly purchased these corrosive substances to commit suicide. The quantity of the ingested material ranged between 10 ml and 150 ml with a mean of 42.6 ml ± 33.2. Table 3 describes the distribution of various commonly used corrosive materials.

**Table 3:** Distribution of various corrosive materials used in corrosive ingestion.

Types of corrosive agents	Frequency	Percentage
Agricultural use (Acid)	8	3.9%
Industrial use (Acid)	16	7.8%
Bathroom cleaner (Acid)	129	62.6%
Caustic soda (Alkali)	9	4.4%
Bleach	44	21.4%
Mode of availability		
Home	166	80.6%
Workplace	22	10.7%
Purchased	18	8.7%
Approximate quantity of ingested material (ml)		
10-50	166	80.6%
51-100	33	16.0%
101-150	7	3.4%

The shortest hospital stay was one day, and the longest one was 60 days. The mean duration of first hospital stay was 11.8 days ± 11.1. Hematemesis and respiratory distress (requiring tracheostomy) were reported in 168 (81.6%) and 13 (6.3%) cases, respectively. While dysphagia and hoarseness of voice were chief complaints of 188 (91.3%) and 48 (23.3 %) patients, respectively. Esophagus and oropharyngeal area were the most common sites which sustained the corrosive injury, whereas corrosive injury to duodenum was least frequent (34.5%). The detailed information regarding clinical presentations of corrosive ingestion patients is given in Table 4.

**Table 4:** Clinical presentations of corrosive ingestion patients.

Features	Frequency	Percentage
Dysphagia	188	91.3%
Hematemesis	168	81.6%
Odynophagia	163	79.1%
Nausea	157	76.2%
Mouth burn	119	57.8%
Abdominal pain	97	47.1%
Sialorrhea	104	50.5%
Hoarseness of voice	48	23.3%
Stridor	13	6.3%
Duration of first hospital stay (Days)		
1-10	140	68%
11-20	38	18.4%
22-40	18	8.7%
45-60	10	4.9%

## Discussion

Corrosive ingestion is associated with the potentially catastrophic presentation and long-standing complications. It causes significant morbidity, mortality and prolonged hospital stay, resulting in huge economic burden to poorly resourced health system of developing countries [14]. Study of the epidemiology of corrosive intake is important from a preventive viewpoint. But the available medical literature about corrosive material ingestion is quite controversial and inconclusive. Different studies show different demographic characteristics of corrosive ingestion patients. The current study discovered that 80.1% cases of corrosive ingestion belonged to the population of 15-30 years age group. It is in contrast to the previous studies conducted in Taiwan and America [15, 16]. In a study by Chen et al., patients older than 40 years of age constitute 55.2% of the total sample size. Twenty-one percent were above 65 years of age and only 8% of cases were reported in the age group of 18 years and below [14]. While, in America, 50-80% incidences of corrosive ingestion occur in children [16-18]. Another research reports that the incidence among children in India and Nigeria is 15% and 25%, respectively [19]. These findings show that the incidence of corrosive intake varies significantly in similar age groups of different countries. It is necessary to explore the factors which are responsible for such variation. The present study shows that females are more likely to suffer from corrosive injuries than males. This is similar to the results of a study conducted in Taiwan in which the incidence rate of corrosive intake in females was 3.3–6.0 per 100,000 population compared to 3.0–5.5 per 100,000 population in males. Such findings are also similar to studies of Republic of Macedonia and Turkey [20, 21]. Consequently, it can be deduced that female subjects show higher intentions of suicide than men. According to our study, most subjects ingested the corrosive material which was already available at home for cleaning and washing purpose. Usually, females are more involved in cleaning and washing as compared to men, so they have easy and frequent access to these corrosive materials which are used as toilet cleaners and laundry bleaches. Thus, during emotional outburst women can easily use such agents to commit suicide. This explains the high incidence of corrosive ingestion in the female gender. So, the control on the availability of liquid corrosives at homes and increased emotional support for women with suicidal intention may prevent impulsive ingestions.

There is dire need to determine the exact nature and chemical constituents of various corrosive materials which are frequently ingested in a particular geographic area. As these different corrosive substances exert their injurious effects by entirely different chemical reactions. The extent of tissue damage is quite distinct and variable depending upon the chemical nature of corrosive substances [12]. Many studies have reported the specific names and relative frequency of commonly ingested corrosive materials [7]. For example, a Turkish study reports that hydrochloric acid and sodium hypochlorite were used in 13 (35.1%) and 24 (64.9%) ingestions, respectively. But in our study, we failed to gather information about the exact names and chemical constituents of various corrosive substances. We observed quite improper and inadequate labeling system on the containers of corrosive products. The manufacturers just wrote their trade names, but the information about specific chemical constituents was not given. So we categorized the corrosive materials on the basis of their use in different fields of life. It was done with an intention to explore the commonly misused corrosive products and the field of life in which “corrosive abuse” was more common. We observed that acids used as bathroom cleaner and laundry bleaches were used in the most cases of corrosive ingestion with a relative frequency of 129 (62.6%) and 44 (21.4%), respectively. It is in contrast to the results of studies conducted in Western countries where alkali material accounts for most of the ingestions [7]. But the findings of an Indian study conducted by Zargar et al. are consistent with our study which reports the use of different types of acids in all cases [22]. This also calls for efforts between medical experts, manufacturers, legislators, and law enforcing agencies to try to develop safer chemicals to be used as bathroom cleaners and laundry bleaches in our country. Moreover, the containers should be labeled with appropriate information regarding contents of the containers. Appropriate and adequate legislation should be done to ensure that only safer agents are available in the market and therefore at homes.

Similarly, in this Indian study by Zargar et al., the approximate volume of acid consumed was 50-200 ml, which is concordant with our findings (10-150 ml) [22]. We found that incidence of corrosive ingestion was surprisingly low in patients with past history of depression. This low incidence may be attributed to psycho-social support, counseling sessions, and antidepressants which they received for the treatment of their mood disorder. Perhaps, this behavioral therapy enabled them to cope with the socio-economic and mental stress so they did not commit suicide. But further studies are needed for the thorough exploration of this aspect.

The clinical presentation of corrosive ingestion varies greatly and the initial presentation usually does not give adequate information about the severity of the damage. During this work, we found that symptoms related to gastrointestinal system predominate compared with the symptoms of the respiratory tract. The two different studies by Vezakis et al. also support the findings of our study [23, 24]. This low rate of corrosive injury to respiratory tract is possibly by the dint of the highly efficient protective pharyngeal-glottic mechanism. Probably this mechanism prevents the corrosive substance to reach respiratory tract (especially the lower airway).

**Figure 1:**
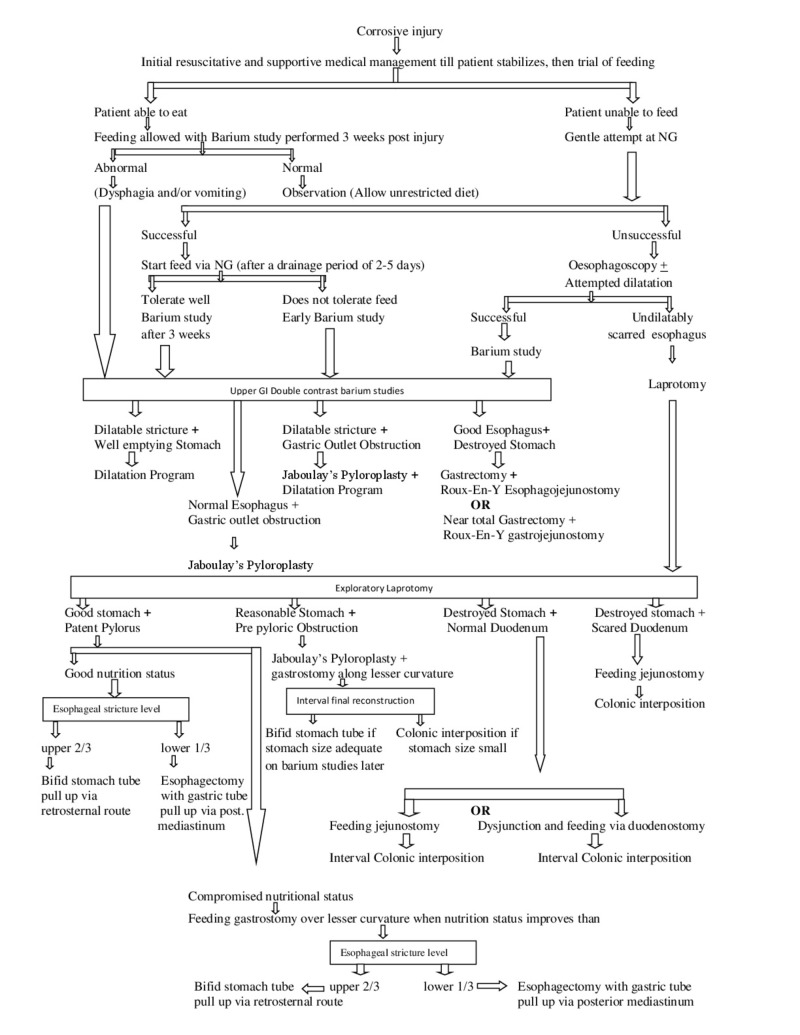
Treatment algorithm for caustic ingestion patients.

The mean hospital stay was 11.8 days ± 11.1 in our study. While the shortest hospitals stay was one day. Out of 206 patients, 25 (12.1 %) were discharged uneventfully just after conservative management. The oropharyngeal area, esophagus, stomach, and duodenum were quite normal in 17%, 1%, 29.1% and 65.5% patients, respectively. Whereas the grade 1 corrosive injury to the oropharyngeal area, esophagus, stomach, and duodenum was seen in 103 (50.0%), 106 (51.5%), 78 (37.9%) and 43 (20.9%) patients, respectively. These findings are close and comparable with the results of studies by Caganova et al. and Zargar et al. [22, 25]. This underlines the need to review the indication of emergency upper GI endoscopy in first 24 hours after corrosive ingestion. The authors are of the view that asymptomatic and patients with minor corrosive injury to aero-digestive tract do not need emergency upper GI endoscopy. Two different studies by Bird et al. and Katz and Kluger have also concluded that in asymptomatic patients emergency endoscopy is quite unnecessary [26, 27]. All they need is careful observation, after which they can be discharged safely on proton pump inhibitors and antacids. Later on, such patients can be followed up for the assessment of complications. We propose that only patients with grade 2 and 3 corrosive injury require intensive care management as most of the complications are associated with these grades of corrosive injury [28]. This strategy of avoiding unnecessary interventions will save the resources of the overburdened health system in developing countries. Based on our observations, we propose a treatment algorithm for the effective management of corrosive ingestion. Figure 1 contains the details of this treatment algorithm.

This model is a beautiful example of evidence-based medicine and can be adopted for the efficient management of critically ill patients of developing countries where the incidence of corrosive ingestion is quite high and the health facilities are limited.

## Conclusions

The management of corrosive injuries requires a multidisciplinary approach and a good coordination between different specialists such as toxicologists, oto-rhino-laryngologists, upper GI and thoracic surgeons, gastroenterologists, psychiatrists, and psychologists. Underprivileged teenager females of rural areas are more likely to ingest corrosive materials with suicidal intention. In most of the corrosive ingestions, household cleaning products are used. The high incidence of corrosive ingestion is due to the illiteracy, poorly resourced health systems, easy availability and unregulated control on the production of corrosive substances. The clinical presentation of corrosive ingestion varies greatly. The symptoms related to gastrointestinal tract predominate while the respiratory symptoms are less common. To reduce the incidence of corrosive ingestion, risk factors and groups at risk should be defined. Appropriate educational programs should be arranged to increase the awareness about the debilitating effects of corrosive ingestion. Enforcing regulations for the manufacturers of household cleaning products can significantly reduce the incidence of this potentially fatal condition.
